# RETRACTION: Our Experience With Propranolol for Infantile Hemangioma

**DOI:** 10.1111/srt.70294

**Published:** 2025-11-15

**RETRACTION**: Y. Wu, P. Zhao, W. Song, W. Lu, T. Dai, and L. Wang, “Our Experience with Propranolol for Infantile Hemangioma,” *Skin Research and Technology* 29, no. 4 (2023): e13310, https://doi.org/10.1111/srt.13310.

The above article, published online on 22 April 2023 in Wiley Online Library (wileyonlinelibrary.com), has been retracted by agreement between the authors; *Skin Research and Technology*; and John Wiley & Sons Ltd. The article was accepted for publication following an insufficient peer review process that does not meet the standards of the journal's peer review policy. Furthermore, several inconsistencies between data presented in tables and results discussed in the text were identified. Accordingly, the article has been retracted.

